# Learning and Reusing Quadruped Robot Movement Skills from Biological Dogs for Higher-Level Tasks

**DOI:** 10.3390/s24010028

**Published:** 2023-12-20

**Authors:** Qifeng Wan, Aocheng Luo, Yan Meng, Chong Zhang, Wanchao Chi, Shenghao Zhang, Yuzhen Liu, Qiuguo Zhu, Shihan Kong, Junzhi Yu

**Affiliations:** 1 State Key Laboratory for Turbulence and Complex Systems, Department of Advanced Manufacturing and Robotics, College of Engineering, Peking University, Beijing 100871, China; wqf@stu.pku.edu.cn (Q.W.); luoac@stu.pku.edu.cn (A.L.); yan.meng@pku.edu.cn (Y.M.); kongshihan@pku.edu.cn (S.K.); 2 Tencent Robotics X, Shenzhen 518057, China; chongzzhang@tencent.com (C.Z.); wanchaochi@tencent.com (W.C.); popshzhang@tencent.com (S.Z.); rickyyzliu@tencent.com (Y.L.); 3 Institute of Cyber-Systems and Control, College of Control Science and Engineering, Zhejiang University, Hangzhou 310027, China; qgzhu@zju.edu.cn; 4 Science and Technology on Integrated Information System Laboratory, Institute of Software, Chinese Academy of Sciences, Beijing 100190, China

**Keywords:** quadrupedal robots, reinforcement learning, motion imitation, variational autoencoder

## Abstract

In the field of quadruped robots, the most classic motion control algorithm is based on model prediction control (MPC). However, this method poses challenges as it necessitates the precise construction of the robot’s dynamics model, making it difficult to achieve agile movements similar to those of a biological dog. Due to these limitations, researchers are increasingly turning to model-free learning methods, which significantly reduce the difficulty of modeling and engineering debugging and simultaneously reduce real-time optimization computational burden. Inspired by the growth process of humans and animals, from learning to walk to fluent movements, this article proposes a hierarchical reinforcement learning framework for the motion controller to learn some higher-level tasks. First, some basic motion skills can be learned from motion data captured from a biological dog. Then, with these learned basic motion skills as a foundation, the quadruped robot can focus on learning higher-level tasks without starting from low-level kinematics, which saves redundant training time. By utilizing domain randomization techniques during the training process, the trained policy function can be directly transferred to a physical robot without modification, and the resulting controller can perform more biomimetic movements. By implementing the method proposed in this article, the agility and adaptability of the quadruped robot can be maximally utilized to achieve efficient operations in complex terrains.

## 1. Introduction

The ultimate goal of biorobotics engineers and scientists is to draw inspiration from biological organisms in nature and to achieve lifelike biomimetic motion by mimicking their behaviors, ultimately contributing to human production and development [1,2]. Quadruped robots are designed to imitate quadrupedal creatures such as dogs and horses [3,4]. With early researchers exploring their mechanical structures, existing quadruped robots share similar forms and designs. They typically have two motors at the hip joint of each leg, one motor at the knee joint, and a total of 12 degrees of freedom. Examples of quadruped robots with similar structures include SpotMini (Boston Dynamics) [5], Cheetah 3 (MIT) [6], ANYMal (ETH) [7], A1 (Unitree) [8], and Lite 2 (DEEP Robotics) [9]. In the case of mechanical convergence, the focus of research in this field lies in achieving smoother movements and accomplishing different tasks for these robots. Currently, some motion controllers such as MPC [10] and whole-body control (WBC) [11] for quadruped robots based on dynamic models have achieved good motion effects. However, model-based controllers require the manual design of motion gaits and involve a significant amount of parameter tuning during controller development [12]. This contradicts the process of biological creatures learning motion in nature, and artificial gaits and motion patterns find difficulty in reaching the level of expertise seen in natural organisms.

End-to-end reinforcement learning methods can automate the learning process of robotic motion, requiring only sensor information as input to generate joint motor movements. Owing to the potentially irreversible damage to the robot and its surrounding environment during the trial-and-error process of reinforcement learning training, the majority of training processes are conducted in simulation environments, followed by the transfer of the trained policies to real quadruped robots. Using an imitation learning framework, refs. [13,14] learned an imitation controller with a similar style from the motion data of biological dogs. However, the work only accomplished an imitation task without obtaining a viable motion controller. Researchers have utilized reinforcement learning to train motion control in complex terrains [15,16] and achieved more robust results compared to traditional methods, which demonstrates the robustness of reinforcement learning methods. Climbing stairs, as an application scene that showcases the unique charm of quadruped robots, has received extensive attention from researchers. Strategies trained using reinforcement learning [17,18] can also efficiently accomplish this task. Furthermore, hierarchical reinforcement learning methods have shown excellent performance in completing challenging tasks such as climbing [19], playing football [20], and chasing games [17]. The above-mentioned methods have achieved robust motion controllers through reinforcement learning, but the majority of these methods utilize pre-defined foot trajectory optimization settings or central pattern generators (CPG) [21], without leveraging the expert knowledge of biological dogs in the natural world.

To enable robots to learn basic motions and higher-level tasks more effectively, a natural idea would be to simulate the learning process of humans or other organisms. Taking inspiration from the human growth process, we first learn to crawl on the ground, then gradually lose reliance on our hands, and learn to stand on two feet. After becoming proficient in walking, we slowly learn to run and jump. As we reach a certain age, we acquire all these basic motion skills, and then, we learn other advanced movements according to our interests, such as kicking a football or playing basketball. It is worth noting that when learning advanced skills, we do not deliberately focus on basic abilities like walking or running. Moreover, in humans and other organisms, there is often no clear boundary between different motion skills when performing higher-level skills. They can transition naturally. Inspired by the process of human growth, designing hierarchical reinforcement learning methods becomes natural: training a motion controller at the bottom level of the reinforcement learning hierarchy to equip the quadrupedal robot with basic motion abilities, and training a task controller at a higher level to enable the quadrupedal robot to complete user-defined tasks.

This article uses imitation learning to teach a quadrupedal robot the realistic gait of a biological dog. Firstly, various motion capture data of gaits are collected from a biological dog’s movements. Inspired by the field of computer animation, a variational autoencoder (VAE) structure is introduced during online reinforcement learning training. The biological dog motion capture data are compressed into a bounded hyperspherical distribution in the latent space. Through the fine-tuning of imitation rewards, the quadrupedal robot can gradually learn various gaits and movement styles of the biological dog and possesses the ability to smoothly transition between gaits. Finally, based on the frozen parameters of the lower-level motion controller, training is directly conducted to map user-defined tasks to the latent space, ensuring that the quadrupedal robot can learn higher-level tasks while retaining the motion skills and style of a biological dog. In brief, the main contributions of this article can be concluded in twofold: (1) we utilize imitation learning to achieve the imitation of biological dog locomotion via a quadrupedal robot; (2) building upon the imitation task, we introduce a hierarchical reinforcement learning framework inspired by biological learning processes for higher-level task acquisition.

The rest of this article is organized as follows. The main methods are presented in Section 2. The experimental results are detailed in Section 3. Conclusions and future work are summarized in Section 4.

## 2. Materials and Methods

This section first introduces the construction method of treating the robot learning tasks as reinforcement learning problems, as well as the redirection process of converting motion capture data of a biological dog into usable reference trajectories for a quadrupedal robot. The specific network structure, state space, action space, termination conditions, and reward settings are also detailed in this section. The design principles of the VAE latent space distribution are also described at the end of this section.

### 2.1. Reinforcement Learning Problem Formulation

Some states and external environmental information of the robot are unobservable due to the unavailability of relevant sensors. The process of learning the movement style of biological dogs and high-level tasks can therefore be abstracted as a partially observable Markov decision process (POMDP). The environment’s states at time *t* are represented by $s_{t}$, and the observable states at time *t* are represented by $o_{t}$. In the training process using simulated environments, all states can be obtained through simulators, so in practice, training is carried out using complete observations $s_{t}$, while the information gap between partial observations $o_{t}$ and $s_{t}$ will be learned through a teacher–student structure. The current agent’s policy network outputs an action $a_{t}$ with probability $\pi (a_{t}|s_{t})$ based on the observed state $s_{t}$. The transitions to the next state $s_{t+1}$ with probability $P(s_{t+1}|s_{t},a_{t})$, and the interaction between the agent and the environment results in a reward $r_{t}$. The above-process can be represented by a transition $\left(s_{t},a_{t},s_{t+1},r_{t}\right)$. By repeating the above process, a trajectory $\tau =\{\left(s_{0},a_{0},s_{1},r_{0}\right),\left(s_{1},a_{1},s_{1+1},r_{1}\right),\cdots ,\left(s_{T-1},a_{T-1},s_{T},r_{T-1}\right)\}$ can be obtained. The goal of reinforcement learning is to find a policy π that maximizes the expected return:

(1) $$J\left(\pi \right)=E_{\tau ∼p(\tau |\pi )}\left[\sum_{t=0}^{T-1}\gamma^{t}r_{t}\right]$$

where *T* represents the length of each episode, $\gamma^{t}\in \left[0,1\right)$ is the discount factor, and p(τ|π) represents the probability of generating a certain trajectory τ when using policy π. It is not difficult to observe that this probability can be calculated using a formula:

(2) $$p\left(\tau |\pi \right)=p\left(s_{0}\right)\Pi_{t=0}^{T-1}p\left(s_{t+1}|s_{t},a_{t}\right)\pi \left(a_{t}|s_{t}\right)$$

where $p(s_{0})$ represents the probability of initializing the state as $s_{0}$. When calculating the expected return, it is not necessary to use this probability. Instead, the expectation is estimated by sampling actual data using the Monte Carlo approximation method.

The policy gradient method is a commonly used approach for optimizing the parameters of policy networks. Our policy network utilizes the Proximal Policy Optimization (PPO) [22] algorithm, which has shown good performance on many challenging tasks. The value network is optimized using the Temporal Difference (TD) algorithm, which incorporates multi-step accumulated discounted return and temporal difference updates. Additionally, the General Advantage Estimation (GAE) [23] method is used for the computation of the advantage function in the policy gradient.

### 2.2. Overview of Network Architecture

**Figure 1:**
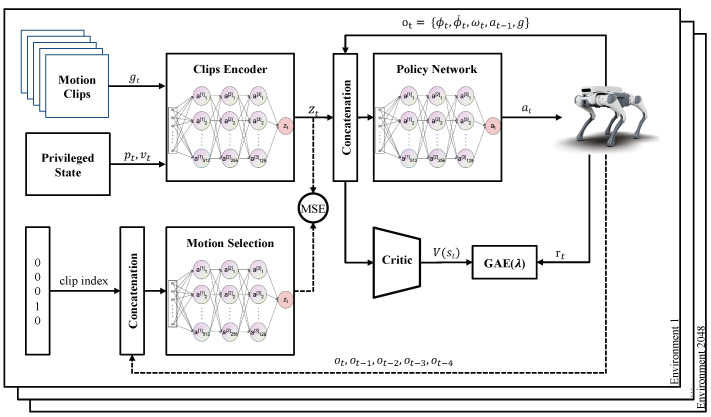
Motion imitation task network architecture. The clip encoder receives motion clips $g_{t}$ and privileged observations as inputs and outputs latent variables. These latent variables are then concatenated with the observations and fed into the policy network, which outputs the target positions $a_{t}$ for 12 joint motors. The motion selection network determines the latent variables corresponding to the desired gait based on historical observation information and the user-inputted gait ID.

**Figure 2:**
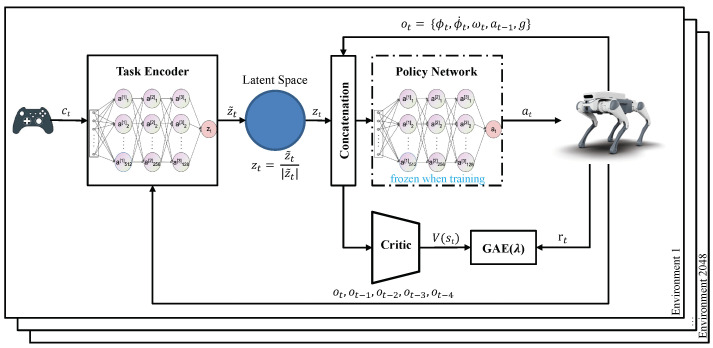
High-level task training network architecture. The parameters of the policy network remain consistent with the policy network obtained through imitation task training. The task encoder receives user velocity command and historical observation information and outputs latent variables distributed in hyperspace.

Referring to Figure 1 of the first imitation task, the clip encoder encodes privileged information and reference motion trajectory clip information $g_{t}$ into latent variable $z_{t}$. The policy network takes the latent variable $z_{t}$ and the historical observation $O_{t}$ as inputs and generates a 12-dimensional output $a_{t}$ of joint positions to make the motion of the quadrupedal robot as consistent as possible with the reference motion trajectory. To achieve gaits or motion mode transitions in the quadrupedal robot based on user commands, a motion selection encoder is trained simultaneously with the training of the clip encoder and the policy network. This network takes a one-hot variable representing the gait or motion mode and historical observations $O_{t}$ as inputs, and its output is designed to be as consistent as possible with the output of the clip encoder. The aforementioned complete framework constitutes a student–teacher structure. The motion selection encoder and the policy network work together during actual deployment, where the user can provide a one-hot variable to imitate the corresponding gait. All three networks mentioned are three-layer perceptrons. From the task of reusing the lower-level policy network to train the upper-level velocity tracking controller, the network framework is illustrated in Figure 2. The lower-level policy network is the same as in the imitation task, and its parameters remain fixed in this task. The task encoder is a three-layer perceptron that takes a three-dimensional velocity command input and historical observation $O_{t}$ input and outputs the corresponding latent variable $z_{t}$.

### 2.3. Motion Retargeting

The motion style of the quadrupedal robot learned through imitation learning primarily depends on the motion data collected by the motion capture system from the biological dog. Motion capture involves attaching motion capture markers to key parts of the biological dog, such as the hip joints and paws, and guiding the biological dog to perform various desired actions to collect motion data of these markers in different motion gaits. The mechanical structure of the quadrupedal robot, such as the lengths of the thigh and calf, as well as the position of the limbs, differs significantly from that of the biological dog. Therefore, the collected gait data from the biological dog cannot be directly used and require mapping the positions of key markers to the quadrupedal robot. At each time step *t*, $f_{t}=\left\{p_{t},q_{t},\phi_{t}\right\}\in R^{19}$ uniquely represents the position, orientation, and joint positions of the quadrupedal robot, where the position is represented by the generalized coordinates $p_{t}\in R^{3}$ of the quadrupedal robot, the pose is represented by the quaternion $q_{t}\in R^{4}$, and the joint positions are represented by the angles in radians $\phi_{t}\in R^{12}$. In the world coordinate system, the position of each key point in the motion capture data is represented as ${\hat{x}}_{i}\left(t\right)$, and the positions of the two shoulder joints and two hip joints of the quadrupedal robot can be viewed as functions $x_{i}\left(p_{t},q_{t}\right)$ of the robot’s position $p_{t}$ and quaternion $q_{t}$. Inspired by [13], we can obtain the sequence of quadrupedal robot poses corresponding to a certain segment of motion capture data by directly minimizing the squared error:

(3) $$\arg\min_{p_{0:T},q_{0:T}}\sum_{t}\sum_{i}\left|\right|{\hat{x}}_{i}\left(t\right)-x_{i}\left(p_{t},q_{t}\right){\left|\right|}^{2}+{\left(\bar{q}-q_{t}\right)}^{T}W\left(\bar{q}-q_{t}\right)$$

where the regularization term in the equation above is introduced to make the obtained pose of the quadrupedal robot more stable. Here, $\bar{q}$ represents the desired default pose of the quadrupedal robot, and *W* is a diagonal matrix representing the regularization coefficients. After obtaining the optimized pose of the robot’s torso, the inverse kinematics can be used to calculate the motor joint angles required to achieve the same position for the robot’s feet relative to their corresponding hip or shoulder joints, based on the motion capture data. So far, the motion capture data sequence $x_{0:T}=\left\{{\hat{x}}_{0},{\hat{x}}_{1},\cdots ,{\hat{x}}_{T-1}\right\}$ is transformed into the reference motion trajectory sequence $f_{0:T}=\left\{f_{0},f_{1},\cdots ,f_{T-1}\right\}$. By performing operations on consecutive elements of the reference motion trajectory sequence, the reference motion velocity sequence of the robot $f_{0:T}^{v}=\left\{f_{0}^{v},f_{1}^{v},\cdots ,f_{T-1}^{v}\right\}$ can be computed, where $f_{t}^{v}=\left\{v_{t},\omega_{t},\dot{\phi }\right\}\in R^{18}$.

### 2.4. State Space

This article mainly accomplishes two tasks: (1) achieving imitation of different motion clips and allowing for switching between different motion clips with given commands, and (2) learning higher-level tasks while retaining the imitated motion style of a biological dog. The observation and reward settings for these two tasks differ slightly.

For the first imitation task, inspired by [24], the future reference motion trajectory $g_{t}=\left({\hat{f}}_{t+1},{\hat{f}}_{t+2},{\hat{f}}_{t+15},{\hat{f}}_{t+50}\right)\in R^{76}$ at time step *t* is used as an input to the policy network and value network, where ${\hat{f}}_{t+50}$ corresponds to the reference pose and reference motor position 1 s in the future (with a control frequency of 50 Hz). During the training process, both the policy network and value network can indirectly observe the complete state $S_{t}=\left\{s_{t},s_{t-1},s_{t-2},s_{t-3},s_{t-4}\right\}$ through the encoder and hidden variables, where $s_{t}=\left\{q_{t},p_{t},a_{t-1},g_{t}\right\}\in R^{76}$, and $a_{t-1}$ denotes the action performed at the previous time step. When the policy network is deployed in practice, only partial parameters of $S_{t}$ can be observed, denoted as $O_{t}=\left\{o_{t},o_{t-1},o_{t-2},o_{t-3},o_{t-4}\right\}$, where $o_{t}=\left\{q_{t},\omega ,\phi ,\dot{\phi },a_{t-1}\right\}\in R^{43}$. The unobserved information is referred to as privileged observation, and how to handle the missing privileged information during deployment will be detailed in the network structure section.

For the second task, we take the implementation of a velocity controller as an example, where the policy network can execute corresponding actions based on the given velocity commands. In this task, an additional user-defined command $C=\left\{v_{x},v_{y},\omega_{z}\right\}\in R^{3}$ will be added to the observations $s_{t}$ and $o_{t}$, where the three elements, respectively, represent forward velocity, lateral velocity, and yaw angular velocity.

### 2.5. Action Space

The action $a_{t}\in R^{12}$ output by the policy network, multiplied by the scaling factor α, represents the offset value relative to the default joint angles $\phi_{0}$. In other words, the target angles for the 12 joints are obtained as $\phi_{d}=\phi_{0}+a_{t}\times \alpha$. The motors operate in position mode, and the motor torque is calculated by a lower-level PD controller with fixed gains ($k_{p}=20$ and $k_{d}=0.7$):

(4) $$\tau^{dof}=k_{p}\times \left(\phi_{d}-\phi \right)+k_{d}\times \left({\dot{\phi }}_{d}-\dot{\phi }\right).$$

### 2.6. Reward Function Design

There is a significant difference in the rewards for the two tasks. For the imitation learning task, the learned policy network should strive to replicate the reference motion obtained through redirection. Thus, the reward can be designed by calculating the error between the reference motion and the motion generated by the policy network. The Gaussian reward function is a commonly used reward function in reinforcement learning, given by $r=\exp\left[-E/\sigma \right]$, where *E* represents the error that needs to be minimized and σ is a parameter that adjusts the reward curve. To control the imitation effect more precisely, we set a threshold for the acceptable imitation error. When the imitation error is equal to this threshold, the reward is assigned a heuristic value of 0.2 (with a maximum score of 1). For joint position error, each joint can tolerate an error of $10^{\circ }$, resulting in a threshold $T^{\phi }$ of 12 joints’ cumulative error: $T^{\phi }=\sum_{i=1}^{12}{\left[10\times \frac{\pi }{180}\right]}^{2}\approx 0.365$. The standard deviation for the joint position error reward can be calculated as $\sigma^{\phi }=\frac{T^{\phi }}{-\ln0.2}=0.227$. For body position error, the acceptable error is 10 cm. For body orientation error, the acceptable error is $10^{circ}$. The end effector can tolerate a displacement of 0.5 cm. Their threshold $T^{p},T^{q},T^{e}$ calculation method is similar to $T^{\phi }$, which will not be elaborated here. Due to the difficulty of fully tracking the velocity, the standard deviation for the velocity error reward curve is determined based on the actual error range.

The importance of different reward terms varies. For imitation tasks, if the joint angles are accurately imitated, it is possible to achieve the same motion trend as the reference trajectory. Therefore, the term $r_{t}^{\phi }$ is crucial for successful imitation, and its weight $w^{\phi }$ should be set relatively high. The weights and specific calculation methods for other terms are tabulated in Table 1, and the target value will be denoted with a hat superscript. Additionally, there may be slight vibration in the limb movements of action clips after redirection. Without modifying the redirection algorithm, adding a penalty term $r_{t}^{\ddot{\phi }}$ for motor acceleration can also restrict the imitation of abnormal movements. The overall reward of imitation learning tasks can be obtained by multiplying each reward by its corresponding weight and summing them together:

(5) $$r_{t}=w^{\phi }r_{t}^{\phi }+w^{\dot{\phi }}r_{t}^{\dot{\phi }}+w^{e}r_{t}^{e}+w^{pose}r_{t}^{pose}+w^{vel}r_{t}^{vel}+w^{\ddot{\phi }}r_{t}^{\ddot{\phi }}.$$

In training for the high-level velocity tracking task, the parameters of the policy network remain fixed, and only the high-level encoder network is updated. The constraints on the latent space ensure that the encoder output of the high-level task can generate actions that comply with a biological dog’s motion style, so there is no need to set rewards related to motion style. The quadrupedal robot should autonomously select appropriate motion clips or their combinations for execution according to the velocity commands. The accuracy of tracking velocity commands $v_{xy}$ and $\omega_{z}$ is the most important for the current task. To maintain smoothness of motion, some rewards and penalties need to be set for motor torque $\tau^{dof}$, acceleration $\ddot{\phi }$, vertical velocity $v_{z}$, and angular velocity in the xy-direction $\omega_{xy}$. The weights and calculation formulas for all reward components of the velocity tracking task are provided in Table 2. Similarly, the reward for this task can be calculated by taking the weighted average:

(6) $$r_{t}=w^{v_{xy}}r_{t}^{v_{xy}}+w^{\omega_{z}}r_{t}^{\omega_{z}}+w^{v_{z}}r_{t}^{v_{z}}+w^{\omega^{xy}}r_{t}^{\omega^{xy}}+w^{\ddot{\phi }}r_{t}^{\ddot{\phi }}+w^{\tau }r_{t}^{\tau }.$$

### 2.7. Termination Condition

In the first imitation learning task, setting the termination condition is very important. At the beginning of the training, the quadrupedal robot has not mastered any motion skills, and random actions can easily cause it to fall. If the episode is not terminated promptly, it will result in a large amount of ineffective exploration by the quadrupedal robot, which will affect the training efficiency. Similarly, when the simulated quadrupedal robot deviates too much from the position or posture of the reference motion, the quality of the collected training data is not good, so it is necessary to set a termination condition for the position and posture deviation. The termination condition for position and posture deviation should not be set too small; otherwise, in the initial exploration process, it will be difficult for a quadrupedal robot to learn the correct way to start moving. In our tests, setting a maximum position error of 0.5 m and a maximum axis angle error of $45^{\circ }$ for posture has a good training effect. Since a large number of explorations at the beginning of the training are incorrect, it is helpful to set a course for the quadrupedal robot to make its learning curve smoother. In this course, the quadrupedal robot first learns which leg to take the first step with, then learns how to take a better first step, and finally gradually tries to take a step with the second leg until it learns a complete gait cycle. It is only necessary to dynamically modify the maximum episode length based on the current training iteration count to achieve the desired course setting. The setting of the maximum episode length at the initial moment $L_{start}$ is critical. If it is set too small, the quadrupedal robot will spend a lot of time learning actions that will not be used in the future, which is reflected in the reward curve as an initial decline. Based on experience, we set the initial maximum episode length to $L_{start}=33$ steps (0.66 s); the maximum episode length at the end of the course is $L_{end}=1000$ steps (20 s). The entire course training will last for $S_{tot}=3\times 10^{7}$ steps, and the maximum episode length during the intermediate process is calculated according to the following formula:

(7) $$L_{step}=\left[1-{\left(\frac{S}{S_{tot}}\right)}^{3}\right]\times L_{start}+{\left(\frac{S}{S_{tot}}\right)}^{3}\times L_{end}.$$

### 2.8. Variational Autoencoder and Latent Space

Without imposing any constraints on the latent space, the latent variables outputted by the encoder are unbounded. The unbounded latent space poses significant challenges for exploration in high-level tasks. Inspired by [25], the latent variables $z_{t}=\frac{{\tilde{z}}_{t}}{|{\tilde{z}}_{t}|}$ from the clip encoder, motion selector, and task encoder are normalized before being used as inputs to the policy network. This aforementioned constraint ensures that the latent variable distribution resides on a hypersphere with a radius of 1. By exploring high-level tasks within this hypersphere latent variable, it becomes easier to discover meaningful latent variables. Otherwise, the policy network may output actions that lead to unnatural behaviors in the quadrupedal robot.

## 3. Experiments and Analysis

This section presents the training settings and experimental platform for quadrupedal robot learning and showcases the test results pertaining to the imitation task and the velocity tracking task. Regarding the imitation task, some visualization analyses were conducted on the data distribution of the reference motion trajectory in latent space.

### 3.1. Experimental Setup

In order to improve the efficiency of data sampling during personal computer training in interactive simulation environments, this study utilizes the GPU-based massively parallel simulation environment, Isaac Sim [26]. In this environment, thousands of agents can be simultaneously activated for data sampling, whereas traditional CPU-based simulation environments such as Pybullet [27] only allow for data sampling with a maximum of a few dozen threads. Based on our empirical tests, training in Isaac Sim can reduce training time by approximately one to two orders of magnitude, depending on the specific task. To reduce development costs, we utilize the recently introduced Isaac Orbit [28], a modular environment for robot learning based on Isaac Sim. This modular environment defines common data structures and operational interfaces for quadruped robots, allowing for easy deployment of quadruped robots in the simulation environment with minor modifications.

All simulation training was conducted on a PC equipped with an Intel I7-11700 CPU and an Nvidia RTX 2060S GPU (Santa Clara, CA, USA). Completing the training tasks outlined in this article required approximately 5 to 10 h. Each reference motion clip in the dataset is approximately 1 s in length, with a total length of approximately 20 s, including pace and trot gaits as well as some turning gaits. The maximum episode length was set to 20 s (1000 steps), and each reference motion trajectory could be cyclically executed until the maximum episode length was reached or termination conditions were met. The control frequency of the policies in the simulation iwa set to 50 Hz. Due to the presence of sensor noise, the measurements from sensors fluctuate around the true values. In order to mitigate the impact of sensor measurement accuracy on learning performance, different noise ranges were set based on the characteristics of each sensor in this work to enhance the learning performance. Additionally, due to discrepancies between simulation parameters (ground friction and ground restitution) in the simulated environment and the real environment, as well as modeling errors in the robot model (robot mass and center of mass position), a significant sim-to-real gap could occur. Therefore, this work utilized domain randomization techniques, where simulation parameters and robot attributes were randomly selected during training. This ensures that the trained model is effective within a certain range of parameters, while real-world parameters are treated as a special case. The specific parameters for randomization are detailed in Table 3. In order to enhance the disturbance resistance of the policy network, during the training phase, a velocity offset within the XY plane was applied to the robot’s center of mass every 15 seconds. The range of velocity offset was [0,1].

Based on the hardware resources of the graphics card, it has been tested that a maximum of 2048 quadrupedal robot can be trained simultaneously in Isaac Sim. Given the large number of agents, to maintain a reasonable batch size in the order of magnitude range, it is necessary to decrease the steps of each agent interacting with the environment in a batch of data. According to the test results in [26], if the number of consecutive steps per agent in a batch of transitions is too small, it will make converging the training difficult. Therefore, in this study, the number of consecutive steps for which each agent interacts with the environment was set to 48 to ensure the convergence of the algorithm. Each batch of data will be used for 5 epochs, with the batch data being randomly divided into 4 mini-batches for optimization within each epoch. The gradient descent utilizes the Adam optimizer [29] with an adaptive learning rate from 1 × 10${}^{-5}$ to 1 × 10${}^{-2}$. For detailed hyperparameters, please refer to Table 4.

We conducted simulation experiments using the Jueying Lite2 quadrupedal robot produced by the company DeepRobotics. It has a body length of 55 cm and a width of 32 cm, with a total weight of 12 kg and a single leg weight of 1.12 kg. The lengths of the thigh and shank are 18 cm and 19 cm, respectively, while the radius of the foot cushion is 2 cm. The peak torque of the two hip joints is 14 Nm, and the peak torque of the knee joint is 23 Nm. The range of motion for the hip swing joint is $\pm 25^{\circ }$, the range of motion for the hip flexion joint is $204^{\circ }$, and the range of motion for the knee joint is $120^{\circ }$. All experimental results are presented in the Nvidia Isaac Sim simulation environment with a simulation frequency of 200 Hz, and a control frequency of 50 Hz. All neural networks were implemented using PyTorch [30].

### 3.2. Motion Imitation Experiment

We utilized the open-source motion capture data of biological dogs from [31]. Based on the motion velocity and patterns, the collected data can be classified into several types, including pace, trot, left turn, and right turn. The pace gait has a relatively slow speed of around 0.8 m/s, with the characteristic that the limbs on the same side move simultaneously. The trot gait has a speed of approximately 1.4 m/s, and its characteristic is that the diagonally opposite limbs move simultaneously. To increase the size of the dataset and enhance the robustness of the data, we performed a left–right mirror transformation on the redirected reference motion trajectories.

**Figure 3:**
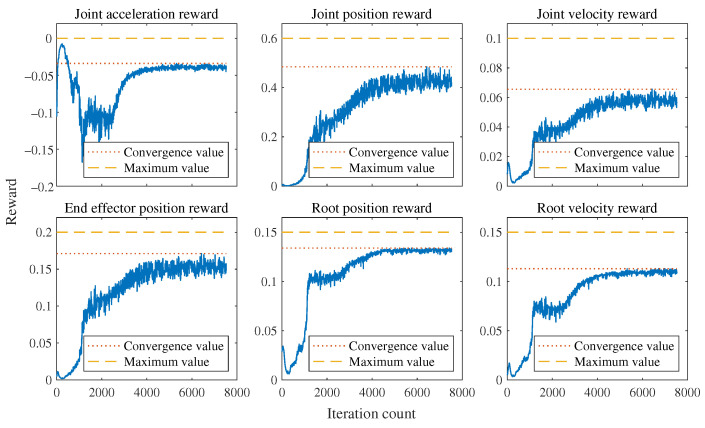
Motion imitation task rewards curve. A higher value for the reward is preferred regardless of its positive or negative nature. The joint acceleration reward is utilized to ensure smoothness in joint movements, while the remaining rewards are employed to maintain similarity between the motion generated by the policy network and the reference motion as much as possible. The maximum value for the joint acceleration reward is 0, while the maximum values for the other rewards correspond to their respective weights of 0.6, 0.1, 0.2, 0.15, and 0.15.

**Figure 4:**
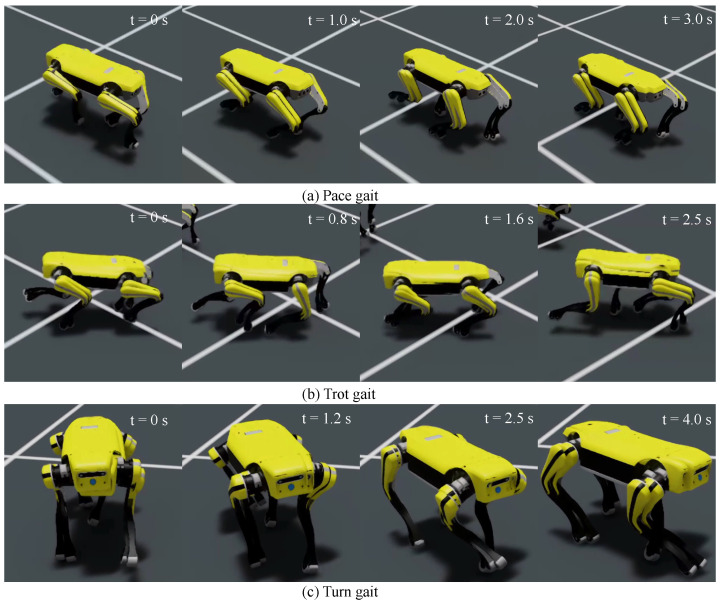
Demonstration of the reference robot and simulated robot in Isaac Sim, exhibiting minimal positional error.

**Figure 5:**
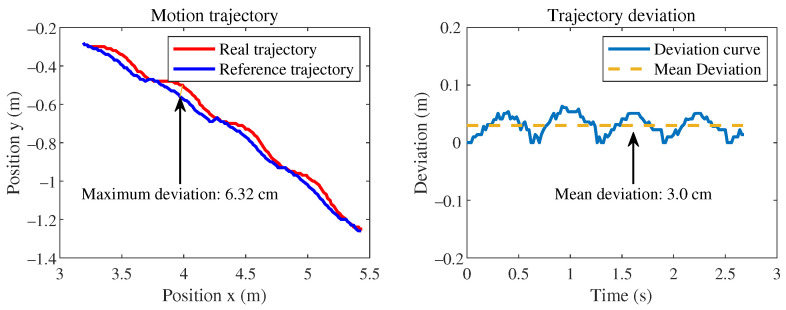
The reference motion trajectory created by concatenating 4 motion clips of pace gait and simulated trajectory and trajectory deviation curve. The maximum deviation distance of the motion imitation process is 6.32 cm, while the average deviation distance is 3.0 cm.

When using the MLP policy network to imitate individual motion segments, it is important to note that the redirected motion capture data are feasible only at the kinematic level and may not be achievable at the dynamic level. Therefore, reinforcement learning attempts to learn a motion trajectory near the reference motion trajectory that satisfies dynamic feasibility. The rewards set during the learning process are determined based on the error between the reference motion trajectory and the actual simulated motion. The average reward of all steps within a certain period is used to evaluate the quality of the current policy network’s output actions. Due to the nature of dynamic feasibility, the rewards find difficulty in converging to the set maximum value. Therefore, training is considered successful when the rewards converge near the highest value. From Figure 3, it can be observed that the rewards for joint positions, body positions, and foot positions can converge to approximately 90% of the maximum score, while the rewards for joint motor velocities and body velocities converge to around 40% and 65% of the maximum score, respectively. From the perspective of imitating the motion style of biological dogs, the designed network has accomplished the task. When both the reference motion trajectory and the simulated robot are loaded in the simulation environment (see Figure 4), it can be observed that they are generally well aligned without significant discrepancies (see Figure 5).

**Figure 6:**
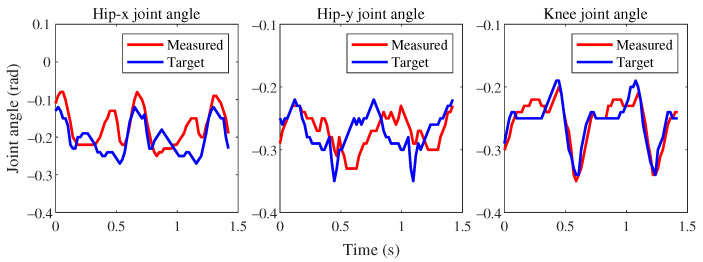
The angle errors of two joints around the x-axis and y-axis of the reference robot and the simulated robot’s hip, as well as the knee joint, over two cycles. Due to the constraints of dynamics feasibility, the hip-x and hip-y joints will have a maximum tracking error of 0.12 rad ($5^{\circ }$).

In the first imitation task, we visualized the reference motion trajectory redirected from the biomimetic dog motion capture data to the Jueying Lite2 and compared it with the imitative motion generated by the trained policy network in an intuitive manner. From Figure 6, it can be observed that the knee joint angle error between the reference robot and the simulated robot is extremely small. There is a slight phase error in the hip-Y joint angle between the reference robot and the simulated robot, but the amplitude is generally consistent. The hip-X joint exhibits slight discrepancies in the curve at certain positions, but the overall difference is minimal. Overall, the learned policy network can imitate the specified gait type of the user, but due to the existence of feasible solutions in dynamics, the actual joint angles may not necessarily correspond precisely to the reference joint angles.

### 3.3. Ablation Study for the Design of the Reward Terms

**Figure 7:**
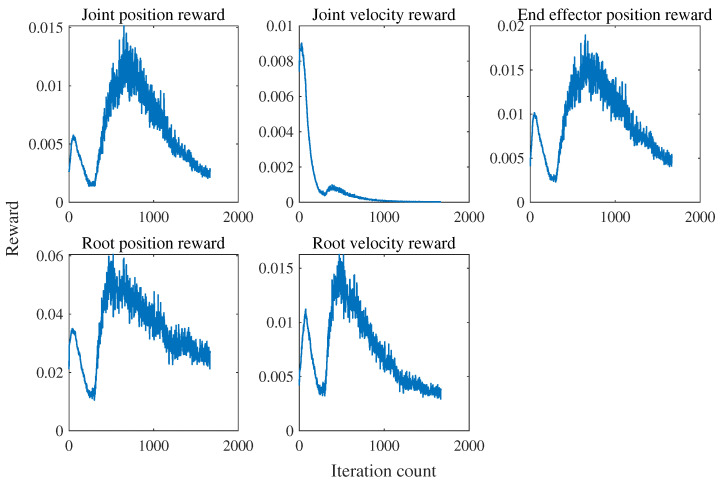
Reward ablation experiment learning curve. The imitation task fails to converge upon removing the joint acceleration reward.

The five reward terms representing the error between the simulated robot and the reference motion trajectory are commonly used in imitation learning for quadruped robots. The reward ablation experiments demonstrated the critical role of the newly introduced reward term for joint acceleration in achieving convergence in imitation tasks. As shown in Figure 7, the omission of the reward term restricting joint acceleration has a significant impact on the learning curve. Imposing certain restrictions on the distribution of the latent space increases the difficulty of convergence in imitation tasks. Without the inclusion of the reward term for joint acceleration, the policy network may generate a series of abrupt and abnormal joint angle outputs, ultimately leading to the inability to learn a policy that resembles the reference motion trajectory.

### 3.4. Analysis of Variational Autoencoder Latent Space

The classic variational autoencoder decomposes the loss function to be optimized into two parts: the reconstruction error between the original data and the generated data, and the KL divergence error between the actual probability distribution of the encoder and the assumed probability distribution. The proportion of these two parts in the loss function is distinguished by multiplying the KL divergence error by the coefficient β. In the imitation task, the reconstruction error can be replaced by the reward from reinforcement learning. By adding the KL divergence error term to the PPO loss function, both the imitation error and the distribution of the encoder can be optimized simultaneously. Before using a regularized latent space distribution, we conducted a series of tests using an encoder that assumes a standard normal distribution in the latent space. To ensure smooth completion of the imitation task, at the beginning of training, β is set to 0 and gradually increases to the maximum value, after which it remains unchanged. Before β reaches its maximum value, the reward has already converged, the purpose of continuing training is to adjust the distribution of the latent space.

Nevertheless, we found that different coefficients β have a significant impact on the convergence of the imitation task. When β is set too large, even if the reward has already converged, the reward curve will gradually decrease due to the adjustment of the latent space distribution in the later stage. When β is set too small, the distribution of the latent space becomes difficult to satisfy the assumption of a standard normal distribution, making it extremely challenging to explore during high-level task reuse of the latent space. It should be noted that the optimal value of β also varies for reference motion trajectories with different data scales, requiring extensive engineering tuning for parameter selection.

To address the above issues, inspired by [25], this study adopts a bounded regularized latent space, which restricts the distribution of the latent space to a hypersphere, avoiding adjustment of its data distribution. It also reduces training time. At the same time, the bounded latent space greatly reduces the possibility of generating unnatural actions for high-level task generation, making exploration easier during high-level reuse.

**Figure 8:**
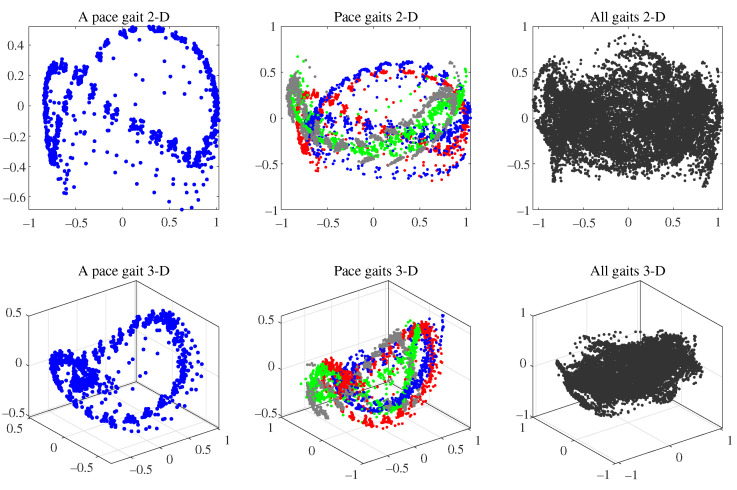
Visualization of latent space of different gaits. The first column represents the visualization of a reference motion clip of pace gait in latent space. The second column represents the visualization of two types of pace gaits (red and blue scatter points) and their mirror extensions (green and gray scatter points). The third column represents the visualization of all motion clips.

To visually represent the distribution of reference motion trajectories mapped onto the latent space, we utilized the PCA algorithm to reduce the dimensionality of the latent space to three dimensions and performed visualization in a 3-D space. The data distributions of the regularized latent space are shown in Figure 8. It can be observed that a reference motion trajectory corresponds to a continuous trajectory in the latent space. Performing symmetric expansion on the reference motion trajectory helps to evenly distribute a certain gait in the latent space. All gaits in the latent space are essentially densely distributed on the hypersphere, facilitating the exploration of high-level tasks.

### 3.5. High-Level Task

**Figure 9:**
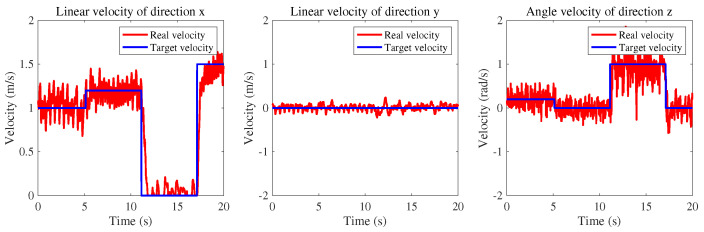
Velocity tracking task curve.

As the foundation for all advanced tasks, the accuracy of velocity tracking is crucial. Validating the effectiveness of this framework can be achieved by accurately tracking the velocity based on imitative tasks, theoretically enabling the completion of more complex tasks. In the velocity tracking task, the velocity instruction is provided by the user’s controller or program, and the task encoder of the quadrupedal robot maps different instructions to different latent variables to select a suitable gait for the current situation. Figure 9 demonstrates the tracking performance under different velocity instructions.

## 4. Conclusions and Future Work

In this work, a hierarchical motion imitation framework for quadrupedal robots is proposed. The contribution of the proposed method lies in achieving precise motion imitation while simultaneously allowing the completion of higher-level tasks based on maintaining the style of the motions. The limitations of this study lie in the fact that the motion capture dataset was conducted on flat terrains, making it challenging for the obtained motion controllers to generalize to complex terrains. Additionally, owing to the dataset lacking comprehensive coverage of all velocity ranges, unnatural gaits may occur when certain velocities are given. Finally, quadruped robots theoretically possess the ability for lateral motion, which can enhance their maneuverability in certain scenarios, but this potential of quadruped robots was not explored in this work.

In future work, further progress will be made in transitioning from simulation to reality. Furthermore, as the data distribution in the latent space is partially influenced by the dataset, adjustments will be made to the dataset size and covered velocity ranges of motion clips. Furthermore, motion data from biological dogs in a 3-D environment, along with artificially generated lateral motion data, will be utilized to augment the existing dataset. The addition of RGB-D data input also holds significant importance for robust motion control in dynamic and complex terrains, which is one of our future endeavors.

## Figures and Tables

**Table 1:** Motion imitation task reward function design.

Reward Term (Weight)	Equation
$r_{t}^{\phi }\left(w^{\phi }=0.6\right)$	$\exp\left[-\left|\right|\hat{\phi }-\phi {\left|\right|}^{2}/0.227\right]$
$r_{t}^{\dot{\phi }}\left(w^{\dot{\phi }}=0.1\right)$	$\exp\left[-\left|\right|\hat{\dot{\phi }}-\dot{\phi }{\left|\right|}^{2}/100\right]$
$r_{t}^{e}\left(w^{e}=0.2\right)$	$\exp\left[-[||{\hat{e}}_{xy}-e_{xy}|{|}^{2}+3\times {\left({\hat{e}}_{z}-e_{z}\right)}^{2}]/0.0248\right]$
$r_{t}^{pose}\left(w^{pose}=0.15\right)$	$\exp\left[-[||\hat{p}-p|{|}^{2}+0.5\times {\left(angle\left(\hat{q}*q^{-1}\right)\right)}^{2}]/0.0441\right]$
$r_{t}^{vel}\left(w^{vel}=0.15\right)$	$\exp\left[-(||\hat{v}-v|{|}^{2}+0.1\times ||\hat{w}-w|{|}^{2})/0.5\right]$
$r_{t}^{\ddot{\phi }}\left(w^{\ddot{\phi }}=-2.5\times 10^{-7}\right)$	$\left|\right|\ddot{\phi }{\left|\right|}^{2}$

**Table 2:** Velocity tracking task reward function design.

Reward Term (Weight)	Equation
$r_{t}^{v_{xy}}\left(w^{v_{xy}}=1.0\right)$	$\exp\left[-\left|\right|{\hat{v}}_{xy}-v_{xy}{\left|\right|}^{2}/0.25\right]$
$r_{t}^{\omega_{z}}\left(w^{\omega_{z}}=0.5\right)$	$\exp\left[-{\left({\hat{\omega }}_{z}-\omega_{z}\right)}^{2}/0.25\right]$
$r_{t}^{v_{z}}\left(w^{v_{z}}=-2.0\right)$	$v_{z}^{2}$
$r_{t}^{\omega_{xy}}\left(w^{\omega_{xy}}=-0.05\right)$	$\left|\right|\omega_{xy}{\left|\right|}^{2}$
$r_{t}^{\ddot{\phi }}\left(w^{\ddot{\phi }}=-2.5\times 10^{-7}\right)$	$\left|\right|\ddot{\phi }{\left|\right|}^{2}$
$r_{t}^{\tau }\left(w^{\tau }=-2.5\times 10^{-5}\right)$	${\left||\tau |\right|}^{2}$

**Table 3:** Domain randomization parameters and observation noise during training.

Parameters	Range [Min, Max]	Unit
Torso Mass	[−3.0, 3.0] + nomial value	kg
Ground Friction	[0.5, 1.25]	—
Ground Restitution	[0.0, 0.1]	—
Root Angular Velocity Obs.	[−0.2, 0.2] + nomial value	rad/s
Projected Gravity Obs.	[−0.05, 0.05] + nomial value	—
Joint Position Obs.	[−0.01, 0.01] + nomial value	rad
Joint Velocity Obs.	[−1.5, 1.5] + nomial value	rad/s

**Table 4:** PPO algorithm training parameters.

Hyperparameter	Value
Discounted Factor γ	0.99
GAE Parameter λ	0.95
Entropy Coefficient	0.01
Value Loss Coefficient	1.0
Clip Parameter	0.2
Learning Rate	1 × 10${}^{-5}$∼1 × 10${}^{-2}$
Optimizer	Adam
Environments Number	2048
Learning Epochs Number	5
Mini Batches Number per Epoch	4
Rollout Steps Number per Environment	48

## Data Availability

Data are contained within the article.
